# Association of personality traits with dental visit procrastination by Japanese university students

**DOI:** 10.1186/s13030-023-00288-z

**Published:** 2023-09-28

**Authors:** Yukitaka Hoshino, Shota Kataoka, Toshihiro Ansai

**Affiliations:** Division of Community Oral Health Development, Kyushu Dental University, 2-6-1, Manazuru, Kokurakita-ku, Kitakyushu, Fukuoka 803-8580 Japan

**Keywords:** Procrastination, Big five personality traits, Illness perception, Delayed dental care

## Abstract

**Background:**

Procrastination is a psychological trait that causes individuals to put off doing things that need to be done. It has recently shown to result in the worsening of symptoms due to delays in seeking medical care. However, it is not clear how perception of dental disease influences dental visits. This study examined the associations of procrastination and personality traits with delayed dental visits for both acute and chronic conditions.

**Methods:**

Of 599 university students queried, the data of 549 subjects (mean age 19.7 years) were analyzed. A general procrastination scale (GPS), the Big Five personality traits, and oral hygiene habits were used for analysis. The participants were asked about illness awareness conditions related to dental disease, perception of pain in the oral region due to acute oral symptoms and chronic symptoms. The participants were asked the number of days until they decided that treatment was required. Based on the bimodal shape of the distribution, those who answered at least eight days for acute or chronic conditions were classified as the procrastination (P) group and the others as the non-procrastination (Non-P) group.

**Results:**

Significant differences in GPS scores were found between the groups for both acute and chronic conditions, with significant differences in the Big Five traits of extraversion, agreeableness, and neuroticism for an acute condition and extraversion, openness, and neuroticism for a chronic condition. There were no significant differences regarding oral hygiene habits between the groups for either condition. Next, using a Bayesian network, the probabilistic causal relations among procrastination, the Big Five traits, and delays in dental visits for both acute and chronic conditions were analyzed. Among the Big Five traits, conscientiousness and neuroticism were directly related to GPS score. Interestingly, agreeableness was directly related to delays in dental visits only for an acute condition and showed a negative effect, while dental student status had a positive effect on delays in dental visits.

**Conclusions:**

The results showed that procrastination and dentistry department are factors that directly influence delays in dental visits, while agreeableness, a Big Five trait, has a negative effect on individuals with an acute condition.

**Supplementary Information:**

The online version contains supplementary material available at 10.1186/s13030-023-00288-z.

## Background

Procrastination is defined as a voluntary and irrational delay of an intended activity despite being aware of the negative effects [1]. It has recently attracted attention as a factor involved in behavior related to health [2]. In addition, procrastination has been shown to be a failure of self-regulation associated with various personal and situational determinants [3], with the main factors personality characteristics and environmental factors related to the task [4]. Research regarding procrastination has been conducted primarily in regard to academics [1, 5, 6], though more recent studies have also pointed out that procrastination is also in the domain of health behavior [7, 8], resulting in proposal of a procrastination-illness model from the perspective of personality and health psychology. This model considers that procrastination influences disease as an outcome by mediating stress and delay in seeking treatment, factors associated with health-specific dilatory behavior [8]. It is defined as the period of time between initial awareness of symptoms and the first visit to a health care provider [9, 10], and has been conceptualized as a multi-step process that includes delays in appraisal, illness, behavior, and scheduling [9].

Several findings regarding the relationship of procrastination with health behaviors have been presented. For example, individuals affected by procrastination tend to have a smoking habit [11], be obese, and have a high body mass index [12]. On the other hand, to the best of our knowledge, only a few studies have examined the association of procrastination with dental or oral health-related behavior [13, 14], which showed that procrastination is negatively associated with attending a dental check-up examination. Furthermore, Shimamura et al. [15] reported that individuals prone to procrastination in childhood had significantly fewer teeth as adults. Nevertheless, the relationship of procrastination with oral health behaviors has not been deeply examined.

The Big Five personality traits [16] are frequently used to assess the effects of procrastination on health behaviors, based on the concept proposed by Suls and Rittenhouse [17] stating that personality traits are one of the three main factors that have a relationship with increased risk of disease. Among the Big Five traits, conscientiousness and neuroticism have been shown to be predictors of procrastination [8]. However, it remains unclear how personality traits influence dental health behavior via procrastination.

A hypothesis was developed stating that individuals affected by procrastination will postpone a dental visit even when aware of acute signs indicating dental disease. To test the hypothesis, we used a model based on the Big Five traits to examine delays in seeking dental care because of procrastination. In addition, the effects of differences in illness perception between acute and chronic symptoms related to dental disease on performing a dental visit were assessed as a secondary outcome.

## Methods

### Aim of study

The purpose of the present study was to assess causal associations of personality traits and procrastination with behavior related to delay from the time of disease perception to decision to visit a dental clinic for acute or chronic conditions indicating dental disease.

### Study design

This study was conducted as a cross-sectional survey of university students in the area of Fukuoka Prefecture, Japan. A total of 599 students were recruited from five departments related to health science at four universities: Fukuoka Dental College in Fukuoka City (n = 59) and Kyushu Dental College (n = 66), Kyushu Women’s University (Department of Nutrition) (n = 231), and Kyushu Kyoritsu University (Department of Sports, Department of Economics) (n = 243) in Kitakyushu City. In addition to students studying dentistry, who would be expected to have a strong association with behavior related to dental health, students majoring in nutrition, sports, and economics were included in consideration of different types of behavior related to dental health, including response to pain in the oral cavity.

**Fig. 1:**
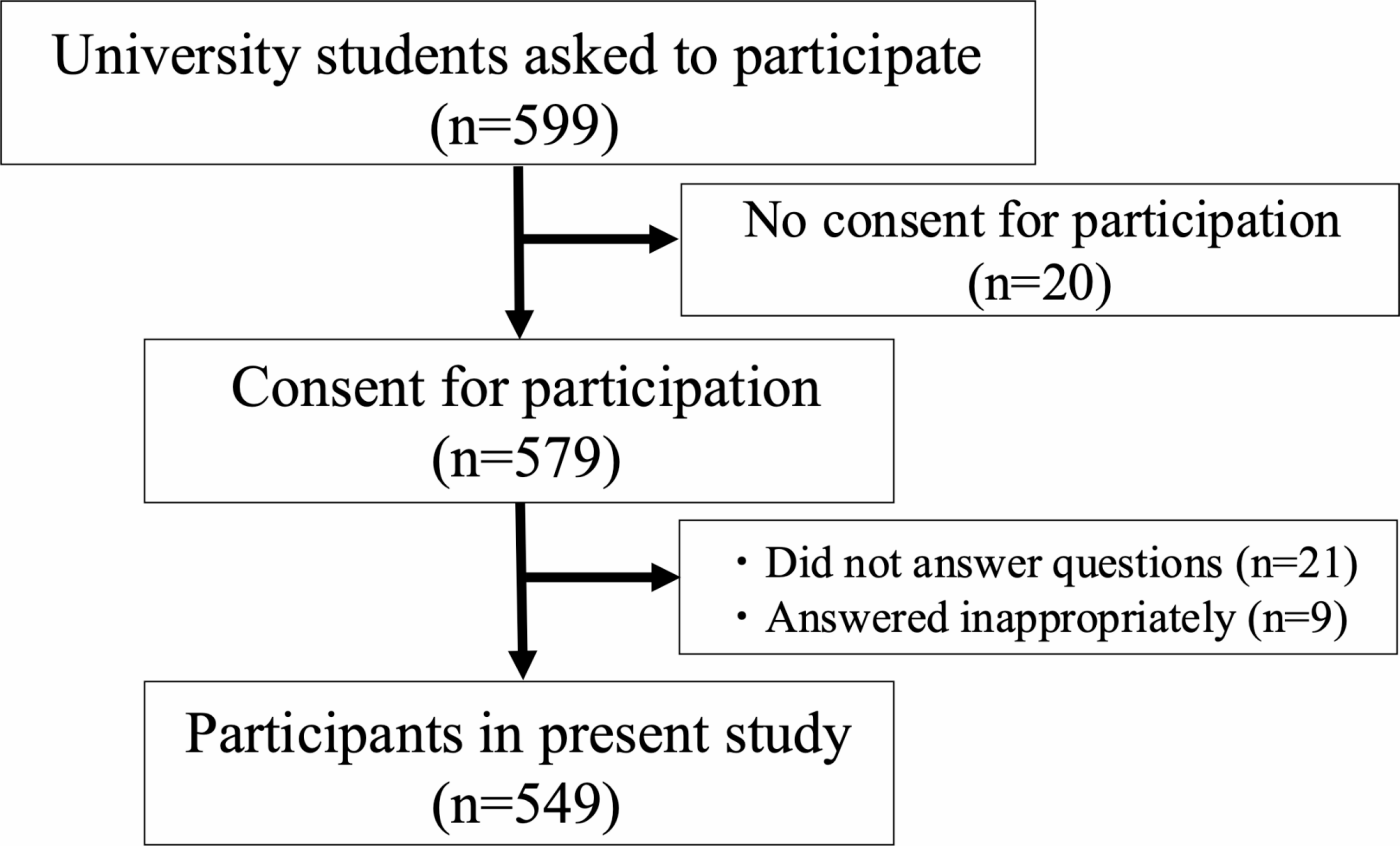
Flow diagram of participant selection

The survey was conducted in a university lecture hall by professors or course instructors of the respective universities using a paper-based, self-administered questionnaire. Before starting the survey, the contents were explained, then after its completion a check for omissions was performed. Students who did not give consent and those who answered inappropriately were excluded, resulting in the data of 549 participants available for inclusion in the analysis (Fig. 1). The study was conducted in accordance with the tenets of the Declaration of Helsinki and approved by the Research Ethics Committee of Kyushu Dental University (Approval No. 22–22).

### Study procedures

The questionnaire included 51 questions regarding gender, aptitude, and personality traits, such as oral hygiene habits, procrastination, and the Big Five personality traits. Following is a sample set of questions. “Suppose you have a regular class examination scheduled in 10 days. Please answer the following two questions. If you were to feel a constant throbbing toothache prior to the examination, would you go to the dentist? If you felt a throbbing toothache only when you bite your teeth prior to the examination, how many days would you wait before going to the dentist?” In other words, two models of illness perception were examined; acute illness perception because of acute pain in the oral cavity and chronic illness perception because of chronic pain during the period before a scheduled test.

### Questionnaires

#### Oral hygiene habits

Items related to oral hygiene habits included daily toothbrushing frequency, number of snacks per day, toothbrushing before bedtime, annual dental checkups, and use of brushing instructions received at a dental office.

#### Procrastination scale

The Japanese version of the general procrastination scale (GPS), originally developed by Lay [6] and then translated into Japanese by Hayashi [18], was used. As noted in the study by Siois (2003), Lay’s GPS is an effective measure for assessing the relationship between procrastination and health behavior. The questionnaire contained 13 related questions, each scored from 1 to 5. For example, “I often find myself performing tasks that I had intended to do several days earlier” and “I often spend time doing other things even when a deadline is approaching”. Cronbach’s alpha coefficient was 0.84, confirming internal consistency.

#### Big five personality traits

The Japanese version of the Ten Item Personality Inventory (TIPI-J) [19] was used. It has been reported to be an appropriate method for time-constrained situations such as seen in school settings [20]. The questionnaire included 10 related questions scored from 1 to 7. Briefly, the score is calculated based on each item and each reverse-scored item. For example, extraversion is calculated by adding (8-item 6) to item 1, with the result indicating a minimum value of 2 and maximum value of 14. Additionally, the questionnaire included the Big Five personality traits, i.e., extraversion, openness, conscientiousness, agreeableness, and neuroticism. For example, for determining extraversion, “I see myself as extraverted, enthusiastic,” and for conscientiousness, “I see myself as dependable, self-disciplined” were used. The TIPI-J is easy to use because of the low number of questions and because it is calculated based on two items for each factor, thus its reliability is confirmed by retest reliability, not by internal consistency [19].

### Statistical analyses

The distribution of behavior based on time from pain awareness to seeking consultation for acute or chronic symptoms showed a bimodal distribution. Therefore, kernel density estimation was performed for both symptoms, which showed “8 days” as the minimum value. Next, the participants were classified into the acute non-procrastination (7 days or less) (Non-P) and acute procrastination (8 days or more) (P) groups for acute symptoms, and the chronic non-procrastination (7 days or less) (Non-P) and chronic procrastination (8 days or more) (P) groups for chronic symptoms. The association of each questionnaire item with examination behavior for acute and chronic conditions as well as early and late phases was evaluated. Questionnaire items that included procrastination and examination behavior were also used. A χ^2^ test was used for analysis of categorical variables and Mann-Whitney’s U test for discrete variables. The significance level was set at α = 0.05.

Bayesian network analysis is a probabilistic model in which qualitative dependencies among random variables are represented by a graph structure and quantitative relationships among individual variables by conditional probabilities. The model is defined by a graph structure that represents dependencies between nodes derived from a set of conditional probabilities. Conditional probabilities with the states of nodes shown adjacent by arrows are propagated one after another through the arrows. The graph structure is then derived from the probabilities of all the nodes. The effective network automatically generated from the data reveals the influence of the relationship between the data items. Bayesian network structure learning using the “bnlearn” package of the R statistical software package was used to examine the probabilistic causal relationship between dental visit behavior and the obtained data. Then, the Bayesian information criterion was used for model fitting and scores were calculated using the hill climbing method. The algorithm was used to rank the network structure based on the scores in regard to the best-fitting model, and increase or decrease in score due to removal of edges. All scores between nodes were calculated and edges between two nodes were generated by the network score when they were directly related. An edge was considered significant if its strength was less than zero. The examiner then evaluated all edges that included that direction. In doing so, illogical edges were “blacklisted” and excluded from the analysis, which improved model fitting and prevented creation of circular structures during the structural learning process. The strength of the edges in the structured Bayesian network was also tested using a bootstrap method with the same R package. The program generated a bootstrap matrix and estimated the network. The process was repeated 1,000 times and probability values were calculated. The factors used in the model were the GPS, assumed to be the top-ranking factor, the Big Five personality traits, and gender, considered to be a basic trait. In addition, any factor for which significant differences were found, i.e., dentistry department was also used in the model.

## Results

### Basic characteristics of participants

Table 1 shows the basic characteristics of the participants. A higher percentage were female (60.8%). Approximately 40% of lived alone and 60% lived with their parents. The most often chosen brushing frequency was two times a day (65.9%) and the most often reported snacking frequency was once a day (48.1%). A majority noted toothbrushing before bedtime every night (76.9%). Furthermore, although 62% did not attend annual dental visits, 58.5% had received brushing instruction at a dental clinic.

**Table 1:** Basic characteristics of subjects (n = 549)

Variable		N (%)
Gender	Male	215 (39.2)
	Female	334 (60.8)
School department	Dentistry	117 (21.3)
	Others	432 (78.7)
Type of residency	Living alone	243 (44.3)
	Living with parents	241 (43.9)
	Student dormitory	56 (10.2)
	Other	9 (1.6)
Daily toothbrushing frequency	Once	31 (5.6)
	2 times	362 (65.9)
	≥3 times	156 (28.4)
Daily snacking frequency	Never	60 (10.9)
	Once	264 (48.1)
	2 times	156 (28.4)
	≥3 times	69 (12.6)
Toothbrushing habits before bedtime	Every night	422 (76.9)
	Other	127 (23.1)
Dental visits (once a year)	Yes	208 (37.9)
	No	341 (62.1)
Toothbrushing instruction received at dental	Yes	321 (58.5)
clinic	No	228 (41.5)
		**Median (IQR)**
GPS		39 (33–46)
Big Five	Extraversion	8 (6–11)
	Openness	8 (7–10)
	Agreeableness	10 (9–12)
	Conscientiousness	7 (6–8)
	Neuroticism	8 (7–10)

***Abbreviations***: ***GPS *general procrastination scale**

### Association between GPS and Big Five, by department

Table 2 shows association between GPS and Big Five based on department, including dentistry. There was a significant association between dentistry and other health science departments in regard to gender and extraversion of the Big Five, no significant associations were found for other personality traits.

**Table 2:** Association between GPS and Big Five by department

Variable		Dentistry	Health Sci 1	Health Sci 2	Health Sci 1 and 2	Dentistry vs. Health Sci 1&2			
		**N (%)**				***P*** ^***a***^			
Gender	Male	65 (55.6)	0 (0)	150 (73.9)	150 (34.7)	< 0.001			
	Female	52 (44.4)	229 (100)	53 (26.1)	282 (65.3)				
		**Median (IQR)**				***P*** ^***b***^			
GPS		40 (34–47)	41.0 (35–47)	37.0 (32–43)	39.0 (33–45)	0.28			
Big Five	Extraversion	8.0 (7–10)	8.0 (6–10)	9.0 (8–11)	8.0 (6–11)	0.03			
	Openness	8.0 (7–10)	8.0 (6–9)	8.0 (7–10)	8.0 (7-9.3)	0.77			
	Agreeableness	10.0 (9–12)	11.0 (9–12)	10.0 (8–11)	10.0 (9–12)	0.84			
	Conscientiousness	7.0 (5–9)	7.0 (5–8)	8.0 (7–9)	7.0 (6–8)	0.92			
	Neuroticism	8.0 (7–10)	9.0 (7–11)	8.0 (7–9)	8.0 (7–10)	0.67			

^**a**^**χ2 test**, ^**b**^**Mann-Whitney U test. *Abbreviations***: ***IQR *interquartile range**, ***GPS *general procrastination scale**

***Dentistry***: **Department of Dentistry**, ***Health Sci 1***: **Department of Nutrition**, ***Health Sci 2***: **Department of Sports and Economics**

### Associations between questionnaire items and the procrastination status of participants with acute and chronic conditions

Table 3 shows oral health-related, GPS, and Big Five traits according to the procrastination status of participants with acute and chronic conditions. The percentages for the P group were significantly higher for members of the school of dentistry as compared to others for both the acute and chronic groups, while there was no significant association of oral hygiene habits with the delay of dental visits for either condition. On the other hand, there was a significant difference in GPS between the P and Non-P groups with acute and chronic conditions. Of the Big Five, significant differences were found for extraversion, agreeableness, and neuroticism for participants with an acute condition, and for extraversion, openness, and neuroticism for participants with a chronic condition.

**Table 3:** Characteristics of participants with acute or chronic condition according to procrastination status

		Acute			Chronic		
Variable		Non-P (n = 350)	P (n = 199)	P *	Non-P (n = 314)	P (n = 233)	P *
		N (%)	N (%)		N (%)	N (%)	
Gender	Male	131 (37.4)	84 (42.2)	0.28	128 (40.6)	87 (37.2)	0.43
	Female	219 (62.6)	115 (57.8)		187 (59.4)	147 (62.8)	
School deparment	Dentistry	55 (15.7)	62 (31.2)	< 0.001	46 (14.6)	71 (30.3)	< 0.001
	Others	295 (84.3)	137 (68.8)		269 (85.4)	163 (69.7)	
Type of residency	Living alone	157 (44.9)	86 (43.2)	0.76	140 (44.4)	103 (44.0)	0.61
	Living with parents	149 (42.6)	92 (46.2)		133 (42.2)	108 (46.2)	
	Student dormitory	37 (10.6)	19 (9.5)		36 (11.4)	20 (8.5)	
	Other	7 (2.0)	2 (1.0)		6 (1.9)	3 (1.3)	
Daily toothbrushing frequency	Once	20 (5.7)	11 (5.5)	0.99	18 (5.7)	13 (5.6)	0.95
	2 times	230 (65.7)	132 (66.3)		206 (65.4)	156 (66.7)	
	≥3 times	100 (28.6)	56 (28.1)		91 (28.9)	65 (27.8)	
Daily snacking frequency	Never	35 (10.0)	25 (12.6)	0.78	34 (10.8)	26 (11.1)	0.99
	Once	172 (49.1)	92 (46.2)		152 (48.3)	112 (47.9)	
	2 times	100 (28.6)	56 (28.1)		90 (28.6)	66 (28.2)	
	≥3 times	43 (12.3)	26 (13.1)		39 (12.4)	30 (12.8)	
Toothbrushing habit	Every night	278 (79.4)	144 (72.4)	0.07	250 (79.4)	172 (73.5)	0.13
before bedtime	Other	72 (20.6)	55 (27.6)		65 (20.6)	62 (26.5)	
Dental visits (once a year)	Yes	138 (39.4)	70 (35.2)	0.36	116 (36.8)	92 (39.3)	0.59
	No	212 (60.6)	129 (64.8)		199 (63.2)	142(60.7)	
Toothbrushing instruction	Yes	206 (58.9)	115 (57.8)	0.86	176 (55.9)	145 (44.1)	0.16
received at dental clinic	No	144 (41.1)	84 (35.2)		139 (44.1)	89 (38.0)	
		**Median (IQR)**		P **	**Median (IQR)**		P **
GPS		38 (33–45)	40 (35–47)	0.007	37 (32–45)	41 (36–47)	< 0.001
Big Five	Extraversion	8.5 (6–11)	8.0 (6–10)	0.025	9.0 (6–11)	8.0 (6–10)	0.005
	Openness	8.0 (7–10)	8.0 (6.5-9)	0.33	8.0 (7–10)	8.0 (6–9)	0.024
	Agreeableness	10.0 (9–12)	10.0 (8–11)	0.002	10.0 (9–12)	10.0 (9–11)	0.05
	Conscientiousness	7.0 (6–8)	7.0 (5–8)	0.19	7.0 (6-8.5)	7.0 (5–8)	0.17
	Neuroticism	8.0 (7–10)	9.0 (7.5–11)	0.010	8.0 (7–10)	9.0 (7–11)	0.009

*χ2 test, **Mann-Whitney U test. *Abbreviations*: ***IQR *interquartile range**, ***GPS *general procrastination scale**

### Bayesian network analysis

**Fig. 2:**
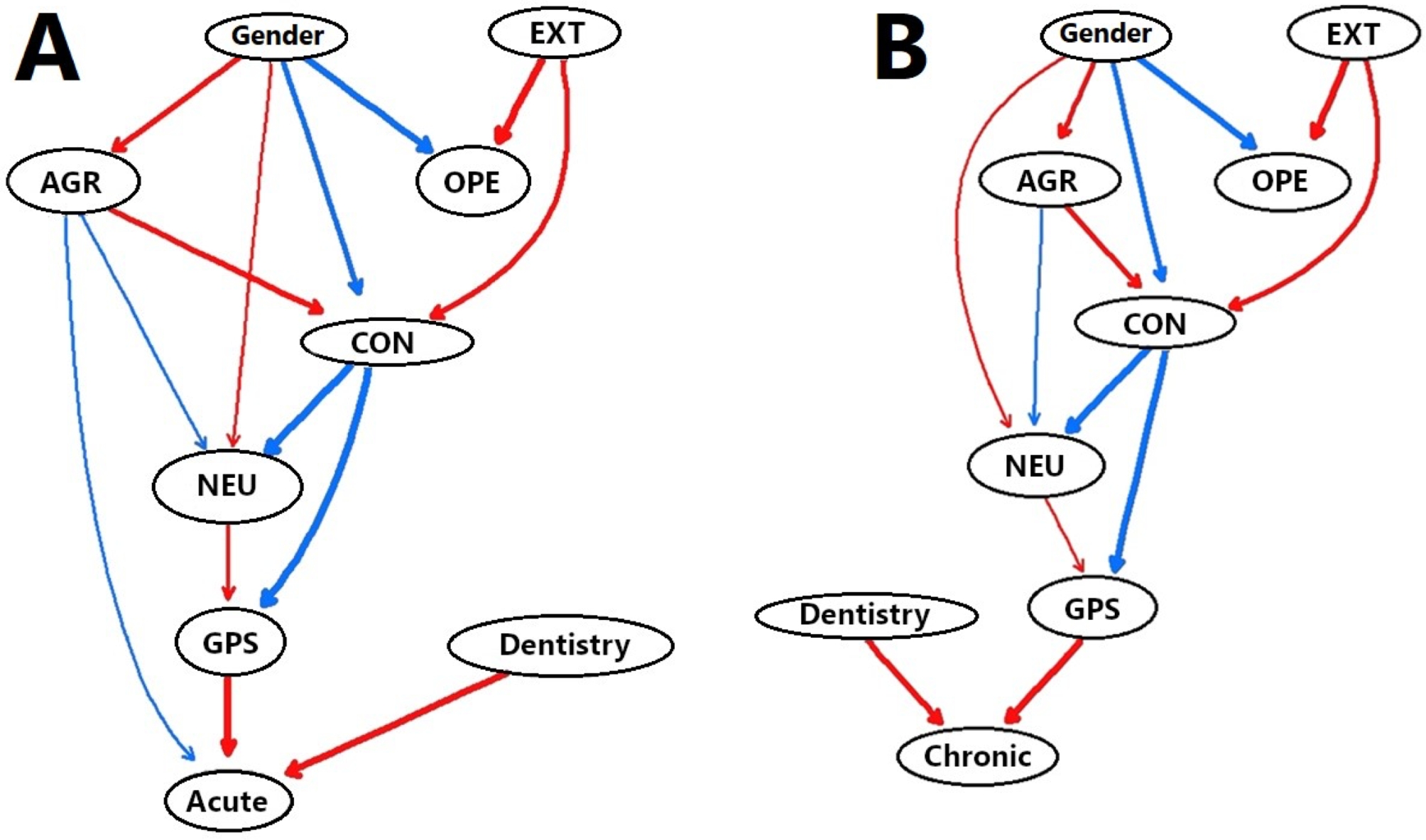
Bayesian network analysis of (**A**) acute and (**B**) chronic conditions. Acute: ref: Non-P, Chronic: ref. Non-P. *EXT* extraversion, *OPE* openness, *AGR* agreeableness, *CON* conscientiousness, *NEU* neuroticism, *GPS* general procrastination scale. Red edges represent positive partial correlations and blue edges negative partial correlations

Bayesian network analysis was conducted to investigate the probabilistic causal associations of the Big Five traits and delays in dental visits because of procrastination. In the network structure derived from the data set, dental visit behavior was set at the lowest level, then edges were drawn from GPS to the dental visit behavior of participants with acute and chronic conditions (Fig. 2A and B), which resulted in a high score for dental visit delay behavior in participants with a high GPS score. The Big Five factors that directly edged GPS were conscientiousness and neuroticism, with conscientiousness having a negative effect and neuroticism a positive effect on GPS.

The Big Five traits placed on higher levels of conscientiousness were agreeableness and extraversion, which had a positive directional effect on conscientiousness. Those associated with neuroticism were agreeableness and conscientiousness, which had negative effects on neuroticism. The edge of extraversion was drawn to openness and conscientiousness, which showed a positive influence on neuroticism. The edges of gender were drawn to the top-ranked factors of agreeableness and neuroticism or conscientiousness and openness, with the former two showing influence in a positive direction and the latter two influence in a negative direction. Furthermore, independent of the Big Five and GPS, membership in a department of dentistry was a factor found to influence delayed dental visit behavior in a positive direction. On the other hand, some differences were found in the structure between acute and chronic conditions. As for the acute condition, agreeableness was directly edged with dental visit behavior and had a negative directional effect (Fig. 2A). In contrast, for the chronic condition there was no direct edge with dental visit behavior (Fig. 2B).

## Discussion

The present study was conducted to examine the associations of procrastination and personality traits with dental visit behavior. The findings showed that procrastination leads to a delay in visiting a dental clinic, while of the Big Five personality traits, agreeableness was directly related to a delayed dental visit by participants with an acute condition. This is the first known report in the field of dentistry that used Bayesian network analysis to do causal estimation of illness perception of dental disease for both acute and chronic conditions.

Few studies have examined the relationship between procrastination and dental visit behavior [13, 14], and those that did found that procrastination had a negative association. In contrast, the present findings indicated no association of procrastination with dental visits (Table 2), possibly because the participants were divided into acute and chronic dental disease groups. Sirois et al. reported that procrastination mediated stress and treatment delay in a study of subjects similar in age to those in the present study [8], and later proposed a procrastination-illness model in which procrastination leads to ill health through mediation of stress and treatment delay [13]. Based on those findings, the authors considered that procrastination may contribute to poor health outcomes through a direct stress-related route as well as an indirect behavioral route. The direct route involves creation of unnecessary stress through procrastination and associated psychophysiological reactivity, which may then lead to changes in immune function that can adversely affect health. On the other hand, they also noted that indirect pathways include the behavioral path and interaction of personality with the environment, possibly resulting in delay of health-protective and promotion of unhealthy behaviors.

Of the Big Five traits, procrastination was associated with conscientiousness and neuroticism for both the acute and chronic conditions. The present results are consistent with previous findings showing a relationship of procrastination with university student academic performance [21]. In addition, bedtime procrastination, a subtype, has been reported to be associated with conscientiousness and neuroticism [22], and high neuroticism and low conscientiousness with poor diet, lack of exercise, and poor physical health [23]. Thus, it is suggested that high neuroticism and low conscientiousness lead to delayed dental visit behavior.

It was evident that the Big Five traits are hierarchically related to procrastination, a novel finding of the present study. Agreeableness and extraversion were positively associated with conscientiousness, located at higher levels of conscientiousness and neuroticism. Also, the present findings indicate that extraversion is associated with delayed dental visits or other unhealthy behavior because of the passage through conscientiousness. Extraversion has been reported to be related to positive aspects such as coping with stress [24], while other studies have found that extraversion may be influenced by aspects of emotional impulsivity [25] and unrealistic optimism [26], and that it is more likely to lead to underestimation of the risk of illness [27]. There is a possibility that such characteristics of extraversion might have led to the present results.

Higher agreeableness has been reported to be correlated with reductions in the risk of Alzheimer’s disease, cardiac mortality, and excess alcohol consumption [28], and also associated with good health status and health behavior. In the present study, three outcomes related to agreeableness were noted. First, it was positively associated with conscientiousness and negatively associated with neuroticism. Second, agreeableness led to delayed dental visits because of procrastination. Third, a direct association of a lower level of agreeableness with delayed dental visits was seen in regard to an acute condition. Findings regarding the relationships of acute health issues, such as headaches, influenza, and digestive issues, with agreeableness have been presented [29], while chronic conditions such as hypertension were shown to be associated with procrastination [30]. However, to the best of our knowledge, no study has been reported regarding a procrastination-health model for the dental disease of persons with acute and chronic conditions. Thus, the reason for those findings cannot be clearly explained and may be due, at least in part, to the dual nature of agreeableness, such as its influence on experiencing positive and negative emotions within the past month [31], and positive and negative correlations with life satisfaction [32].

Among our participants, dental students tended to delay dental visits as compared to students in other departments. Possible reasons for this finding are as follows. First, it has been reported that the oral health of recent dental students shows improvement, with fewer having significant dental caries [33, 34], indicating that many dental students have no experience of severe pain. In addition, a study by Miura et al. [35] that compared dental and pharmacy students found that dental students were more aware of oral health, which may explain why the dentistry department factor affected procrastination. Second, the impact of stress on dental students undergoing a school-related examination should be considered. Results of a systematic review indicated significant stressors related to examinations among dental students [36]. Fear of facing parents after a school-related failure, such as failing an examination or receiving poor grades, was also found to be one of the most stressful factors for dental students in some countries [37, 38], while other reports have noted that dental students show higher levels of stress as compared to individuals in the general population [39, 40]. As a result, they may tend to prioritize exam preparation over a dental visit.

Based on the results of the present study, consideration of practical ways to reduce procrastination during the student period or even younger is required. For example, enhancing self-regulation may be possible. Several cognitive-behavioral methods (e.g., implementation intention imagery) [41] as well as training in self-regulation skills, including stimulus control, goal definition, and time management techniques [42], might also be useful. Shimamura et al. [15] reported that individuals with a strong tendency toward procrastination in childhood had significantly fewer remaining teeth in old age. Also, an investigation of life course studies noted inter-generational transmission of risk factors, including socioeconomic and environmental factors, which are known to be easily passed from parent to child generations [43]. Therefore, useful measures for prevention of procrastination are required, especially during the time when students begin to become socially independent.

This study has some limitations that should be noted. First, because of the cross-sectional design, strong causal relationships cannot be inferred. Second, the participants were enrolled from a limited number of community-dwelling university students and it is not clear whether the current findings can be applied to other young populations. Further studies are needed to test the generalizability of the results. Third, the results of the self-administered questionnaire may have differed as compared to those from individuals with an awareness of actual pain: the participants were asked to imagine acute and chronic pain. Finally, analyses regarding past history of dental disease or whether any of the subjects were currently undergoing dental treatment were not performed. However, the impact on the outcome of this study is considered to be minimal, because the number of subjects who had experienced invasive dental procedures is considered to be limited. Nevertheless, a more detailed investigation is needed in the future. Despite these limitations, the outcomes of the present study are considered to be applicable to dental professionals as well as community, government, schooling, and community healthcare practitioners who promote prevention of dental disease.

## Conclusions

The present findings indicate that procrastination and being a dentistry department student are important factors that directly influence dental visits, as is the involvement of agreeableness, a Big Five personality trait, as a negative factor in individuals with an acute condition.

### Electronic supplementary material

Below is the link to the electronic supplementary material.

Supplementary Material 1

## Data Availability

The datasets analyzed in the current study are available from the corresponding author upon reasonable request.

## Abbreviations

GPS: General procrastination scale
TIPI-J: Japanese version of the Ten item personality inventory
SD: Standard deviation
