# Supplementary material for: Association of personality traits with dental visit procrastination by Japanese university students
Source: Biopsychosoc Med. 2023 Sep 28;17:33. doi: 10.1186/s13030-023-00288-z (PMC10538028; doi:10.1186/s13030-023-00288-z)
Supplement: Supplementary file 1 — Supplementary Material 1 [file 13030_2023_288_MOESM1_ESM.docx]

July 28, 2023

Editor

*BioPsychoSocial Medicine*

To the Editor:

Thank you very much for sending the helpful comments and suggestions from the reviewers concerning our manuscript, entitled “Association of personality traits with procrastination for dental visits in Japanese university students” (BPSM-D-23-00036). After carefully reviewing those, several changes have been made to the text. As a result, we believe that the revised version is greatly improved and now acceptable for publication in *BioPsychoSocial Medicine*.

Changes in the revised manuscript are highlighted in yellow, while our point-by-point responses are included following. We sincerely thank the reviewers for their valuable insights and you for your interest in our work.

We would be grateful if the revised manuscript could be reviewed and considered for publication in the journal.

Yours sincerely,

Toshihiro Ansai, DDS, PhD

Division of Community Oral Health Development

Kyushu Dental University

Kokurakita-ku, Kitakyushu 803-8580 Japan

Fax: +81-93-591-7736

Email: r06ansai@fa.kyu-dent.ac.jp

Following are our replies to the comments from Reviewer I.

Abstract:

1) Methods: 28 line: Why did you divided into 2 groups? Please explain about the criteria for dividing.

- Thank you for this question. On page 9 (line 142) in the original version of the manuscript, it was noted that the distribution showed a bimodal shape. However, based on the good suggestion, additional details have been added to the Methods section in the Abstract of the revised version (page 2, line 28).

2）Results and Conclusions: Why didn't you write about dentistry students, more significance factor rather than the Big five traits?

- Based on the helpful question, some sentences in the Abstract (page 3, line 41) and Conclusion (page 19, lines 322 to 323) sections have been rewritten.

Background:

1) Why did you use the Japanese version of the general procrastination scale and the Japanese version of the Ten Item Personality Inventory (TIPI-J) in this study?

- GPS used in this study is considered suitable for investigating the relationship between procrastination and health behavior, as previously reported by Sirois (2003). TIPI-J was used because it has been reported to be an appropriate method for time-constrained situations such as school settings. Accordingly, some explanatory sentences have been added to the revised Methods section (page 8, lines 133 to 135).

Methods:

1） Study design: Why did you recruiting from five departments including dentistry at four universities?

- Based on the helpful question, some explanation has been added to the revised Methods section in the revised manuscript (page 6, lines 90 to 91).

2） Statistical analyses: Why did you divided into 2 groups? Please explain about the criteria for dividing.

- We appreciate this helpful suggestion. As described above (Abstract, question 1), the distribution of behavior showed a bimodal pattern and the kernel density estimation showed that 8 days was the cutoff value. Some related details have been added to the Statistical analyses subsection in the Methods section (page 9, lines 147 to 148).

3） Procrastination scale: Please explain about the Japanese version of the general procrastination scale (GPS), minimum score 13 and maximum score 65, ordinary person's score. Were these students, especially, dentistry students, highly or lowly?

- According to the report by Hayashi (2007), the mean GP value for Japanese aged 20 to 35 years was 40.47 (SD: 8.22). In contrast, in the present study that was 39.39 (SD: 8.97), and when limited to dental students 40.38 (SD: 9.38). Therefore, the participants in this study (including dental students) did not comprise a population with specific characteristics.

4） Big Five personality traits: Please explain about the Japanese version of the Ten Item Personality Inventory (TIPI-J), scoring methods, minimum and maximum and ordinary person's score. Were these any characteristic feature of these students, especially, dentistry students?

- TIPI-J is the Japanese version of TIPI developed by Gosling (2003). As described in the Methods section, five personality traits were assessed. Details regarding the scoring method (including minimum and maximum values) have been added to the Methods section (page 9, lines 136 to 138).
- The table shown following presents comparisons between the report by Oshio et al. (2012) and the present results for all participants and only dental students.
- As seen in the table, there were significant associations between that previous report and the present study for extraversion, agreeableness, conscientiousness, and neuroticism, with the same trend observed for dental students alone. Thus, our study found no significant differences for the five personality traits were found between dental students and other department students.

|  | Extraversion | Agreeableness | Conscientiousness | Neuroticism | Openness |
| --- | --- | --- | --- | --- | --- |
| Oshio et al. | 7.83 ± 2.97 | 9.48 ± 2.16 | 6.14 ± 2.41 | 9.21 ± 2.48 | 8.03 ± 2.48 |
| This study | 8.34 ± 2.95^＊^ | 10.1 ± 2.03^＊^ | 7.15 ± 2.27^＊^ | 8.60 ± 2.42^＊^ | 8.13 ± 2.38 |
| This study (only dental students) | 7.85 ± 2.91 | 10.0 ± 2.12^＊^ | 7.12 ± 2.32^＊^ | 8.54 ± 2.50^＊^ | 8.13 ± 2.38 |

*Significant association between results presented by Oshio et al. and this study.

5） Statistical analyses: Please explain the distribution of days.

- As noted above (Abstract, Methods; Methods, Statistical analyses), the distribution of behavior showed a bimodal pattern.

Results:

1） Basic characteristics of participants: Please explain correlation GPS and Big Five and Gender, Faculty (Table1 and Table2).

- Thank you for this good suggestion. Accordingly, new Table 2 has been included and several sentences have been added to the Results section in the revised version (page 12, lines 191 to 195).

2） Bayesian network analysis: I think Table 3 and 4 are not necessary in explanation. Please explain about Figure2A and 2B in more detail.

- Tables 3 and 4 have been deleted. Furthermore, some additional explanation regarding Figs. 2A and 2B has been included (page 14, lines 227 to 228).

Discussion:

1） page17, 268-279 line: I don't think your opinion, main factor is stress which was the reason of dental students tended to delay dental visits. I think for example, dental students may know dental diseases and have some treatments for themselves or their instructors. What about do you think about my opinion?

- We sincerely appreciate these valuable comments. In addition to stress-related factors, characteristics of dental students are also discussed. Possible reasons why dental students tended to delay dental visits have been added to the Discussion section (page 17, line 282 to page 18, line 294).

Conclusion：

I think you may write a comment about dental students.

- A description regarding dental students has been added (page 19, lines 322 to 323). Thank you for this good suggestion.

Following are our replies to the comments from Reviewer II.

1） Did you not investigate the subject's past history of dental disease? Does the presence or absence of a history of disease affect the results of questionnaires for dental visits?

- Thank you for these important questions. Unfortunately, no details regarding past history of dental diseases experienced by the participants are available. However, as shown in a report of health examinations of dental students performed at our University (Hikiji et al., 2010), it is considered that the dental students were less likely to have experienced severely painful dental treatment, based on their low DMFT values as compared to the results of the Survey of Dental Disease by the Ministry of Heath, Labour and Welfare, conducted with a group of the same age. Therefore, the impact on the results of responses to the present questionnaire is considered to be minimal. Some explanation in this regard has been added to the Discussion section in the revised version (page 17, lines 282 to 287).

2） Are there any subjects currently undergoing dental treatment?

- We appreciate this important question. Unfortunately, we do not have information regarding whether any of the subjects are currently undergoing dental treatment. Nevertheless, as noted above, it is considered that the impact on the present results was minimal because it is unlikely that any of the subjects received invasive dental treatment for dental caries that caused them severe pain. On the other hand, a detailed investigation that includes history of dental disease and current medical history is needed as part of a future study. Some explanation in this regard has been added to the Discussion section (page 19, lines 312 to 316).

3） It is speculated that those findings are due to a kind of stress. Didn't you compare students from medical and pharmacy faculties, who are under the stress of dentistry as subjects?

- Miura et al. (1989) compared dental and pharmacy students, and reported no significant differences in oral findings or caries treatment experience between those groups. Furthermore, it was stated in that study that dental students are significantly more likely to take care of their own dental health. This was observed in another study presented by Inagaki et al. (2013), which reported differences in number of times for toothbrushing per day as well as use of an interdental brush between dental and pharmacy students. Thus, it is suggested that knowledge and behaviors regarding oral health of dental students are different from those of other undergraduate students. Several related sentences have been added to the Discussion section (page 17, lines 282 to page 18, line 294).

4） Is there a reason why dental students are delayed in seeing a dentist because of the amount of knowledge they have about dental diseases compared to other subjects?

- As mentioned above, dental students seem to differ in regard to amounts of knowledge about dental diseases and oral health as compared to other undergraduate students. This is discussed in the Discussion section (page 17, lines 285 to 286).
